# Association of Phase Angle with Body Composition in Hemodialysis Patients: A Case–Control Study

**DOI:** 10.3390/life15111666

**Published:** 2025-10-25

**Authors:** Selma Cvijetić Avdagić, Petra Kovačević Totić, Karla Kovačević Čorak, Antonija Sulimanec, Karmela Altabas

**Affiliations:** 1 Institute for Medical Research and Occupational Health, 10 000 Zagreb, Croatia; 2 University Hospital Centre Sisters of Charity, 10 000 Zagreb, Croatia; 3 Croatian Institute of Public Health, 10 000 Zagreb, Croatia

**Keywords:** hemodialysis, phase angle, muscle mass, bone mass, fat mass, body composition

## Abstract

Patients on hemodialysis (HD) often experience changes in body composition due to metabolic disorders. Phase angle (PhA) is a marker of tissue integrity and may reflect overall functional condition. This study evaluated body composition and its relationship with PhA in 53 HD patients (27 women, 26 men) over 40 years old, compared with 106 age- and sex-matched healthy controls. Body composition was assessed using bioelectrical impedance analysis (BIA), measuring skeletal muscle mass (SMM), fat tissue, total bone mass (BM), and PhA. HD patients had significantly lower fat mass and PhA than controls (*p* < 0.001). The prevalence of low SMM and BM was higher in patients, though not statistically significant. Sex differences were generally not significant, except for a higher prevalence of low BM in female controls (*p* < 0.001). After adjusting for age and sex, PhA was positively associated with SMM% (*p* = 0.021) and BM (*p* = 0.035) in HD patients only. These results indicate that PhA–body composition relationships differ between HD patients and healthy individuals, highlighting PhA as a potential marker of body composition disturbances in HD.

## 1. Introduction

Patients with chronic kidney disease (CKD) undergoing chronic hemodialysis (HD) experience changes in body composition as a result of the disease itself, the dialysis procedure, and comorbidities [1,2]. Body composition is closely linked to nutritional status and can affect clinical outcomes as well as quality of life [3,4]. Therefore, monitoring body composition in HD patients is important for improving health status and survival.

Current evidence indicates that a low skeletal muscle mass is common in HD patients due to various metabolic disorders, including acid–base and electrolyte imbalances, inflammation, and nutritional deficiencies [5,6]. The reported prevalence of sarcopenia in HD patients aged 40–75 years ranges from 25.9% to 34.6%, depending on the definition used [7]. Osteopenia and osteoporosis are also highly prevalent in HD patients due to abnormal CKD-related homeostasis, elevated phosphate and fibroblast growth factor-23 (FGF-23), and reduced vitamin D and calcium levels, leading to secondary hyperparathyroidism and impaired bone remodeling [8,9].

With regard to obesity, its harmful effects on health are well established; however, epidemiological studies suggest that overweight and obesity may have a protective role in HD patients, as excess weight and nutritional reserves might buffer against acute illness and comorbidities, outweighing the adverse cardiovascular effects of obesity [1,10]. In the general population, even among adults over 40 years of age, the prevalence of obesity, sarcopenia, and osteopenia is high [11,12]. A recent meta-analysis reported that, depending on the classification and cut-off values used, the prevalence of sarcopenia ranged between 10% and 27% in the general population [12].

Phase angle (PhA) is a parameter obtained from bioelectrical impedance analysis (BIA) that reflects the phase shift between voltage and current as an alternating electrical current passes through body tissues and reflects the capacitive properties of cell membranes [13]. Therefore, PhA provides insight into cellular health and body composition: higher values indicate greater cell membrane integrity, higher cell mass, and better overall functional status, whereas lower values suggest cell membrane damage, the loss of cellular integrity, or fluid imbalance [14]. In HD patients, PhA is clinically relevant as it reflects the combined effects of inflammation, fluid balance, and cellular condition—factors closely linked to morbidity and mortality [15]. HD patients frequently experience protein–energy wasting, chronic inflammation, and alterations in body water distribution, all of which affect PhA values. PhA appears to be less affected by hydration status compared with conventional BIA-derived body composition components and may therefore serve as a stable and sensitive marker of cellular health [16,17,18], providing qualitative information that cannot be captured by the quantitative BIA components (muscle mass, fat mass, and body water). Due to these properties, PhA has been recognized as a reliable marker of muscle quality and sarcopenia in HD patients [19,20]. Moreover, as an indicator of tissue integrity, PhA may be effective in predicting survival [21,22]. Chertow and colleagues demonstrated that a low PhA was associated with increased mortality in HD patients, even after adjusting for nutritional status [23]. Several studies have also shown that PhA is independently associated with muscle mass, strength, and sarcopenia, suggesting that it can be a useful predictor for identifying HD patients at risk of sarcopenia [16,18,23,24].

The primary aim of this study was to evaluate body composition in patients undergoing maintenance hemodialysis using bioelectrical impedance analysis (BIA), with a focus on the prevalence of low muscle mass, low bone mass, and obesity, and to compare these findings with those of a generally healthy control population matched by age and sex. A secondary objective was to explore the complementary role of PhA in assessing the functional status of HD patients by examining whether its associations with body composition parameters differ between HD patients and healthy individuals.

## 2. Materials and Methods

### 2.1. Participants

This study included 53 patients (27 women, 26 men) aged >40 years who were on a chronic hemodialysis program at the University Clinical Hospital Sisters of Charity in Zagreb during 2020. Inclusion criteria were a dialysis vintage of 6 months or longer. Exclusion criteria were prescribed immunosuppressive therapy and any acute illness in the previous month.

The control group consisted of 106 subjects stratified by sex and age. Younger and middle-aged participants were recruited among volunteers—employees of the Institute for Medical Research and Occupational Health (IMROH)—and among workers referred to IMROH for their annual medical examination. To achieve accurate age matching in older age groups (>70 years), the control participants were recruited from nursing homes. All such individuals were volunteers who met the same inclusion and exclusion criteria as other controls and were generally healthy, community-ambulatory, and free from acute or debilitating illness at the time of recruitment.

For each HD patient, a control participant of the same sex and closely matched age (±1 years when possible) was recruited from the population. This individual matching strategy was employed to minimize the confounding effects of age and sex on body composition parameters. No exclusions were made based on BMI, in order to reflect the natural distribution of body composition across age groups and to maintain comparability with the HD population, which also included overweight and obese individuals.

Exclusion criteria for all controls included chronic kidney disease, any chronic disease requiring glucocorticoid or immunosuppressive therapy, acute illness in the previous month, significantly reduced mobility, immobility, or mental disorders including dementia. For both patients and controls, additional exclusion criteria were health conditions that prevented bioimpedance measurement (e.g., pacemaker, limb amputation).

### 2.2. Measurements

Body weight and height were measured without shoes in indoor clothing using a digital scale and stadiometer (SECA 877 and 217, Hamburg, Germany, respectively) and body mass index (BMI; kg/m^2^) was calculated. BMI ≥ 30 kg/m^2^ was categorized as obesity, 25 ≤ BMI ≤ 30 kg/m^2^ as overweight, and 18.5 ≤ BMI < 25 kg/m^2^ as normal weight.

Body composition measurement was performed with the bioelectrical impedance device BIA-ACC^®^ (BioTekna^®^, Marcon-Venice, Italy). The parameters of measurement were fat mass (FM; % of body weight); abdominal adipose tissue (AAT; cm^2^); intramuscular adipose tissue (IMAT; % of body weight); fat-free mass (FFM; kg); skeletal muscle mass (SMM; % of FFM) yielding the S-score; total bone mass (BM; kg), yielding the T-score; and phase angle (PhA; °). The S-score and T-score compare a subject’s muscle mass and bone mass to those of a healthy young reference population and are expressed in standard deviations. Low muscle and bone mass were identified based on the S-score and T-score of ≤−1.0 [25]. The measurements of whole-body bone mass (kg) alongside other body composition parameters by the BIA-ACC^®^ device have been validated against DXA [26]. Obesity/adiposity was defined based on total fat mass ≥ 25% for men and ≥32% for women [25]. In patients on HD, C-reactive protein (CRP) and serum albumin data were taken from routine laboratory blood tests.

### 2.3. Statistics

The results are presented as mean ± standard deviation for continuous variables and as percentages for categorical variables. The distribution of variables was tested using the Kolmogorov–Smirnov test. Only BMI and dialysis vintage were not normally distributed in patients and BMI and AAT in controls. Therefore, the difference in mean values of body composition parameters between patients and controls was tested using Student’s *t*-test. The chi-square test was used to compare the frequencies of low muscle mass, low bone mass, and obesity between patients and controls.

The correlation between dialysis duration and body composition parameters was tested using Pearson’s correlation.

The multiple regression model was created with PhA as dependent variable and body composition parameters as predictors, separately for patients and controls. Collinearity between continuous independent variables was determined by calculating variance inflation factor (VIF), using the formula 1/(1 − R^2^). The result was 1.31 for the model with patients and 1.57 for the model with controls, indicating mild collinearity between predictors. Since only the patients had laboratory values for CRP and serum albumin, we did not include those parameters in the regression model. Therefore, the association between CRP and serum albumin with body composition parameters was tested separately with Spearman’s correlation. The level of significance in all analyses was set at *p* < 0.05.

Due to the relatively small number of patients, we performed the post hoc power analysis using G*Power software, version 3.1.9.7. (Universität Kiel, Kiel, Germany) to see whether the sample size was informative in terms of indicating power to detect statistically significant associations observed in the regression model for the patients. The input parameters were the sample size (N = 53) and the number of predictors (7), with an effect size of 0.15 and an alpha error probability of 0.05. The power (1 − β err prob) was 0.871.

## 3. Results

**Figure 1:**
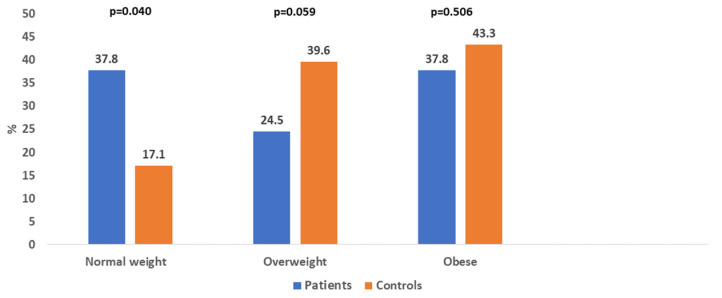
Distribution of normal weight, overweight, and obese patients and controls.

The mean age of the patients was 67.9 ± 11.7 years (range 42–90 years) and 67.4 ± 12.9 (range 41–90 years) for controls. The mean dialysis vintage was 4.2 ± 3.4 years (4.1 ± 3.34 years in women and 4.5 ± 3.7 years in men). According to BMI, 37.8% of patients and 17.1% of controls had a normal weight (*p* = 0.040), 24.5% of patients and 39.6% of controls were overweight, and 37.8% of patients and 43.3% of controls were obese (Figure 1). No patient or control was underweight.

The mean values of FM% and IMAT% were above the reference range, while the PhA was below the reference range in both patients and controls (Table 1). The mean AAT was above the reference range only in controls. Patients had significantly lower FM%, IMAT%, AAT, and PhA than controls (all *p* < 0.001). The mean value of serum albumin in patients was 37.9 ± 5.2 g/L, which is inside the reference range, while the mean value of CRP was increased (11.3 ± 19.6 mg/L; reference limit < 5 mg/L).

Compared with female patients, male patients had a significantly lower FM% (*p* = 0.037) and a significantly higher SMM% and BM (both *p* < 0.001). Male controls had a significantly lower FM% (*p* = 0.026) and a significantly higher AAT (*p* = 0.015), SMM%, BM, and T-score (all *p* < 0.001) than female controls.

A low muscle mass was found in 43.1% of patients and 32.3% of controls. A low bone mass was determined in 45.2% of patients and 42.4% of controls. Obesity, diagnosed according to FM%, was present in 37.7% of patients and 43.3% of controls (shown in Figure 1), which was the same as diagnosed by BMI. Significantly more patients were normal weight than overweight, while significantly more controls were overweight than normal weight (*p* < 0.001 for both). There were no significant differences in the prevalence of obesity, low SMM, and low BM between women and men, both in the patients and in the control group. The exception was a significantly higher prevalence of low BM in female controls compared with male controls (*p* < 0.001).

No significant correlation was found between the duration of dialysis and any of the body composition parameters (BMI, FM%, IMAT%, AAT, SMM%, BM, and PhA).

The results of multiple regression showed that, when controlled by age and sex, PhA was significantly positively associated with SMM% (*p* = 0.021) and BM (*p* = 0.035) in patients, as presented in Table 2. The proportion of the variance in the PhA that can be explained by the predictor variables was 13.8% in patients and 36.4% in controls.

CRP and serum albumin did not significantly correlate with age and with any of the body composition parameters in patients.

## 4. Discussion

We found no significant difference in the prevalence of low muscle and bone mass between patients on chronic HD and age/sex-matched controls. Mean values of bone and skeletal muscle parameters were within the reference values in both patients and controls. However, the controls had significantly higher fat tissue parameters and consequently a higher prevalence of obesity than patients.

The prevalence of low muscle mass in our patients was slightly higher compared with data from a recent meta-analysis [5]. In the studies included in that meta-analysis, in which the definition of sarcopenia was based only on muscle mass [8 of 30 studies], the prevalence of sarcopenia in patients on HD, mean age ranging from 47.5 to 77.5 years, was 34.6%. The prevalence of low muscle mass in our controls was similar to data from another recent meta-analysis that included 52 studies, in which the definition of sarcopenia was also based only on muscle mass [12], with a mean prevalence of 27.5% in participants across a wide age range [<60 years and >60 years].

It is interesting that despite the relatively high prevalence of low muscle mass in our patients, the mean values of SMM% and BM were within the reference range. This points to two possible explanations: We used only the criterion of reduced muscle mass for assessing sarcopenia, without evaluating muscle strength and/or walking speed. In most cases, according to this criterion, the prevalence of sarcopenia is slightly higher compared with the norms proposed by the International Working Group on Sarcopenia [IWGS] or the European Working Group on Sarcopenia in Older People [EWGSOP] [5,12]. Secondly, an equal proportion of our patients were obese or had a normal weight [37.8%], but only one patient among the obese group had a low muscle mass, and only one among the normal weight group did not have a low muscle mass.

These findings suggest that while the overall prevalence of low muscle mass alone was not significantly elevated, individual variations in nutritional and fat status may play a protective role. This has important clinical implications, as it supports the need for individualized risk stratification in HD patients, beyond relying solely on traditional sarcopenia cut-offs. Indeed, some other studies have described that low body fat mass was an independent risk factor for poor survival in dialysis patients [10] and that higher concentrations of total cholesterol are beneficial in these patients due to its effect of eliminating endotoxins that promote chronic inflammation [27]. However, due to metabolic disturbances that can confound body composition measurements in HD patients, it has been proposed that standard cut-off values may misclassify their muscle and bone status. The study by De La Flor et al. proposed novel thresholds for sarcopenia derived from nutritional ultrasonography, which showed meaningful associations with handgrip strength, lean body mass, and phase angle, suggesting better sensitivity and clinical relevance in detecting sarcopenia in this complex patient population [28].

Our results indicated that muscle mass, as well as bone mass, were significant predictors of PhA, which is a known indicator of tissue integrity and consequently of muscle quality and sarcopenia in patients on HD [14]. Several studies have confirmed that PhA is independently associated with muscle mass and that it can be a reliable marker for estimating muscle health in patients with chronic kidney disease [20,24,29].

However, our regression analysis revealed that approximately 36% of PhA variance in the controls could be explained by body composition parameters, compared with less than 14% in our patients. This underscores the complex and multifactorial nature of PhA, which is not determined solely by body composition. Patients on HD are a vulnerable population, usually with multiple medical complications of the underlying disease and therapy, and it is expected that the proportion of body composition parameters as predictors of PhA would be lower than in healthy subjects, as we presented in our results. PhA is influenced by various physiological and pathological factors, including hydration status, inflammation, and functional status—elements that are often altered in the hemodialysis population. Therefore, while muscle and bone mass appear to contribute to PhA variability, they represent only part of the broader clinical picture. In a similar case–control study by Tan and colleagues, patients on HD had a significantly lower PhA than the healthy group, and PhA values were also positively associated with fat-free mass and mid-arm muscle circumference [15]. These results reinforce the potential value of PhA as a complementary marker of functional status but also highlight the need for multidimensional assessment in HD populations. Moreover, although the overall prevalence of low muscle and bone mass did not differ significantly between HD patients and controls, there are distinct patterns in how PhA correlates with specific body composition parameters in these groups. The PhA was significantly lower in HD patients and positively associated with skeletal muscle mass and bone mass, suggesting that PhA reflects multiple aspects of body composition and metabolic disturbances in this population. From a clinical standpoint, our results highlight the importance of including PhA in routine assessments of HD patients, as it provides additional insight into patient status that may not be reflected in conventional body composition parameters. The analytical value of PhA lies in its ability to capture tissue quality and cellular health, offering a more dynamic measure than static estimates of muscle or bone mass. The integration of PhA with standard measures could improve the early detection of nutritional or functional decline, especially in patients who appear well nourished by conventional metrics but may have underlying cellular compromise. This highlights that PhA may serve as a global health marker but should be interpreted alongside direct measurements of muscle and bone mass for comprehensive assessment.

It is known that patients on HD are prone to the development of inflammation, often determined by high levels of CRP [30,31]. It has also been found that obesity and sarcopenia are characterized by low-grade chronic inflammation and that both conditions may be independently associated with inflammatory biomarkers [32,33]. Potentially, chronic inflammation could induce an imbalance between protein synthesis and catabolism, resulting in muscle loss [34]. Although the mean value of CRP in our subjects was slightly increased, we did not find a significant correlation with body composition parameters. As discussed earlier, the good body mass status of our participants may also act as a protector of adequate muscle mass, which is consistent with the assumption that body fat mass and serum cholesterol may reduce endotoxins responsible for chronic inflammation [35]. Moreover, in the same study sample, with a cross-sectional design, we previously found no association between sarcopenia and periodontitis, which is a potential trigger for systemic inflammation [36].

The limitation of this study is the relatively small number of patients. However, we tried to compensate for this with a case–control study design, which allows for the better estimation of associations than cross-sectional models. Moreover, the post hoc power analysis indicated the suitability of the regression model for the patients. However, future studies with larger cohorts and longitudinal designs are warranted to confirm these associations and to explore their clinical relevance over time. Another limitation is the lack of laboratory parameters in the control group, which would have allowed us to compare the relationship between CRP and body composition in both patients and healthy participants. However, we did not observe a significant correlation between CRP and body composition in the patients, which may suggest that their CRP levels were not elevated enough to negatively affect body composition. Also, the relatively low PhA values in the control group may partly reflect the influence of age and body composition, as PhA has been shown to decrease with age and differ between sexes and BMI categories [37,38]. This should be considered when interpreting the generalizability of the findings.

## 5. Conclusions

Although the prevalence of low muscle mass and low bone mass was not significantly different between HD patients and controls, we observed a distinct pattern of association between body composition parameters and PhA in both groups. PhA was significantly lower in patients compared with controls and was positively associated with skeletal muscle mass and bone mass in patients. While PhA is likely influenced by various metabolic and other disturbances in HD patients, it is clear that body composition parameters also play a crucial role in determining PhA and, consequently, the overall health status of patients on HD. These findings emphasize the potential clinical relevance of PhA as a non-invasive and informative biomarker that can complement standard assessments in HD patients. Future clinical protocols might benefit from incorporating PhA alongside traditional body composition analysis to enhance patient monitoring and personalized treatment strategies.

## Figures and Tables

**Table 1:** Age, anthropometry, and body composition in patients on dialysis and controls.

Parameters	Patients (N = 53)	Controls (N = 106)	Reference Range	*p* ^1^
Age (yrs.)	67.9 ± 11.7	67.4 ± 12.9	/	0.805
BMI (kg/m^2^)	28.1 ± 6.3	29.1 ± 5.6	18.5–24.9	0.319
FM%	34.6 ± 8.4	39.5 + 6.9	12–30	<0.001
IMAT%	2.4 ± 0.4	2.7 ± 0.4	<2.0	<0.001
AAT (m^2^)	464.6 ± 225.3	554.9 ± 227.3	<460	<0.021
SMM%	33.0 ± 6.5	32.5 ± 6.6	>30	0.710
S-score	−0.3 ± 1.7	−0.1 ± 1.4	>−1.0	0.282
BM (kg)	3.8 ± 1.0	3.6 ± 1.0	>3.0	0.292
T-score	−0.4 ± 1.1	−0.7 ± 1.0	>−1.0	0.191
PhA (°)	0.5 ± 0.9	1.2 ± 1.0	>3.5	<0.001

^1^ Student’s *t*-test. FM = fat mass; IMAT = intramuscular adipose tissue; AAT = abdominal adipose tissue; SMM = skeletal muscle mass; BM = bone mass; PhA = phase angle.

**Table 2:** Association between PhA and parameters of fat, skeletal muscle, and bone tissue in patients and controls.

	Patients (N = 53)		Controls (N = 106)	
	b ^1^	*p*	b ^1^	*p*
Intercept ^2^	−0.35	0.887	1.88	0.849
Age	−0.02	0.337	−0.04	<0.001
Sex	−0.35	0.524	0.27	0.443
FM%	0.18	0.122	0.04	0.326
IMAT%	−3.63	0.081	0.29	0.473
AAT	−0.00	0.950	−0.01	0.152
SMM%	0.20	0.021	0.05	0.105
BM	1.17	0.035	0.04	0.885

^1^ Coefficient of regression. ^2^ Intercept is an estimated constant term of the regression model (predicted value of the dependent variable when all predictors are zero). FM = fat mass; IMAT = intramuscular adipose tissue; AAT = abdominal adipose tissue; SMM = skeletal muscle mass; BM = bone mass.

## Data Availability

Due to patient confidentiality and ethical restrictions, the datasets are not publicly available but are available from the corresponding author upon reasonable request.

## Abbreviations

The following abbreviations are used in this manuscript:

AAT	Abdominal adipose tissue
BIA	Bioelectrical impedance analysis
BM	Bone mass
BMI	Body mass index
CKD	Chronic kidney disease
CRP	C-reactive protein
FM	Fat mass
FFM	Fat-free mass
HD	Hemodialysis
IMAT	Intramuscular adipose tissue
PhA	Phase angle
SMM	Skeletal muscle mass
